# Pharmacologic Inhibition of Ezrin-Radixin-Moesin Phosphorylation is a Novel Therapeutic Strategy in Rhabdomyosarcoma

**DOI:** 10.1155/2020/9010496

**Published:** 2020-09-09

**Authors:** Austin Proudfit, Nabanita Bhunia, Debasis Pore, Yvonne Parker, Daniel Lindner, Neetu Gupta

**Affiliations:** ^1^Department of Inflammation and Immunity, Lerner Research Institute, Cleveland Clinic, Cleveland, OH, USA; ^2^Department of Pediatric Hematology-Oncology & Bone Marrow Transplantation, Cleveland Clinic, Cleveland, OH, USA; ^3^Translational Hematology and Oncology Research, Taussig Cancer Institute, Lerner Research Institute, Cleveland Clinic, Cleveland, OH, USA

## Abstract

Intermediate and high-risk rhabdomyosarcoma (RMS) patients have poor prognosis with available treatment options, highlighting a clear unmet need for identification of novel therapeutic strategies. Ezrin-radixin-moesin (ERM) family members are membrane-cytoskeleton linker proteins with well-defined roles in tumor metastasis, growth, and survival. ERM protein activity is regulated by dynamic changes in the phosphorylation at a conserved threonine residue in their C-terminal actin-binding domain. Interestingly, ERM family member, ezrin, has elevated expression in the RMS tissue. Despite this, the translational scope of targeting ERM family proteins in these tumors through pharmacological inhibition has never been considered. This study investigates the inhibition of ERM phosphorylation using a small molecule pharmacophore NSC668394 as a potential strategy against RMS. Upon in vitro treatment with NSC668394, RMS cells exhibit a dose-dependent decrease in cell viability and proliferation, with induction of caspase-3 cleavage and apoptosis. siRNA-mediated knockdown of individual ERM protein expression revealed that each regulates RMS survival to a different degree. In vivo administration of NSC668394 in RMS xenografts causes significant decrease in tumor growth, with no adverse effect on body weight. Collectively, this study highlights the importance of the active conformation of ERM proteins in RMS progression and survival and supports pharmacologic inhibition of these proteins as a novel therapeutic approach.

## 1. Introduction

Rhabdomyosarcoma (RMS) is regarded as the most common pediatric soft tissue sarcoma with an incidence of 4.4 cases per million annually [1]. These tumors are of mesenchymal origin and consequently occur in diverse anatomical locations, including the head, neck, trunk region, within the genitourinary tract, and abdominal area [2–5]. The two major histological subtypes of RMS are embryonal (ERMS) and alveolar (ARMS), with ERMS diagnosed more commonly (∼60% cases) compared to ARMS (∼20% cases) [6]. Spindle/sclerosing and pleomorphic RMS account for the remainder of cases [6]. ERMS typically presents in younger children (1–14 yrs.) relative to ARMS; however, both major subtypes are most often diagnosed as pediatric sarcomas [7, 8]. At the molecular level, the presence of chromosomal translocation t(2; 13) (q35; q14), and t(1; 13) (p36; q14), which fuses paired box transcription factors 3/7 (PAX3/7) with forkhead box protein 1 (FOXO1), is associated with ARMS [9]. Interestingly, the histopathological classifications of fusion-negative ARMS have been shown to be molecularly and clinically indistinguishable from ERMS [10]. Frequent genetic alterations in ERMS include loss of heterozygosity (LOH) and imprinting at the 11p15.5 locus, resulting in the overexpression of IGF2 [11–13]. Risk classification of RMS is based on multiple criteria including histological subtype, tumor location, and metastatic potential [6]. Recently, PAX3/7-FOXO1 fusion status has also become an important genetic prognostic indicator, with fusion-positive patients showing worsened outcome compared to fusion-negative patients [14]. Standard treatment regimens for RMS include combination chemotherapy along with surgery and/or radiation, resulting in a five-year event free survival rate of ∼90% for localized, low-risk patients. However, the estimated survival rates drop to ∼70% in patients with intermediate and ∼25% high-risk RMS, underscoring a need for novel therapeutic options [15–17].

Interestingly, studies have shown that human RMS tissues express relatively higher levels of ezrin (*VIL2*) compared to the normal skeletal muscle [18]. Furthermore, the degree of ezrin expression increases with late-stage RMS and has been associated with greater metastatic potential [18, 19]. Suppression of ezrin expression in highly metastatic RMS cell lines was shown to reduce their metastatic potential, while increasing ezrin expression in poorly metastatic RMS cell lines resulted in higher rates of metastasis [18]. Ezrin belongs to the ezrin-radixin-moesin (ERM) family of proteins and consists of a highly conserved *N*-terminal FERM domain, an intermediate *α*-helical domain, and an actin binding C-terminal domain. ERM proteins display conformational switching between closed and open conformations, the latter corresponding to the active form that bridges the plasma membrane and actin cytoskeleton [20]. In their closed conformation, ERMs are unphosphorylated, with their actin binding site masked by the FERM domain [20]. Phosphorylation at its C-terminal threonine residue converts them to an open conformation thereby enabling binding with filamentous actin at the C-terminus and with transmembrane and adaptor proteins at the FERM domain [20]. This crosslinking ability enables a range of cellular processes pertinent to progression and sustainability of tumor cells including survival, proliferation, apoptosis, migration, and invasion [21–26]. Despite the importance of this conserved threonine phosphorylation ERM protein function, it has never been pharmacologically targeted in RMS to assess the effect on tumor growth.

In an effort to identify novel therapeutics approaches for RMS, we hypothesized that pharmacologic inhibition of ERM proteins with NSC668394, a small molecule inhibitor of ERM phosphorylation, will induce antiproliferative and apoptotic effects in RMS.

## 2. Materials and Methods

### 2.1. Cell Lines

RD (CCL-136) and Rh18 are ERMS, whereas Rh30 and Rh41 are ARMS. RD was obtained from ATCC, and Rh18, Rh30, and Rh41 were obtained from Cancer Oncology Group (COG) repository. From a genetic standpoint, RD and Rh18 are PAX-FOXO1 fusion-negative, while Rh41 and Rh30 are fusion-positive. All RMS cell lines were cultured at 37°C with 5% CO_2_. RD and Rh41 were grown in Dulbecco Modified Eagle Medium supplemented with 10% fetal bovine serum (FBS), while Rh18 and Rh30 were grown in Iscove's Modified Dulbecco's Medium with 20% FBS and 1X insulin, transferrin, and selenous acid (ITS).

### 2.2. Viability and Proliferation Assays

Cells were seeded in 12-well or 96-well plates, for trypan blue and MTT assays, respectively, and cells were incubated in complete growth medium containing NSC668394 (1, 2.5, 5, or 10 *μ*M) or vehicle control (DMSO) for 0, 24, 48, 72, and 96 h. Viable cell counts were determined by trypan blue visualization with a hemocytometer. MTT (Sigma-Aldrich, St. Louis, MO, USA) was added to cells at a final concentration of 10% and incubated for 4 h at 37°C. Culture medium was then removed, and formazan crystals produced by reduction of MTT salt were dissolved in DMSO. Absorbance was measured by spectrophotometry at 562 nm.

### 2.3. Apoptosis Assays

RD cells were seeded in 6-well plates and incubated with complete medium containing NSC668394 (5 or 10 *μ*M) or vehicle control (DMSO) for 0, 48, and 96 h. Apoptosis was analyzed using PE Annexin V Apoptosis Detection Kit I (BD Biosciences, Franklin Lakes, NJ, USA) following manufacturer's guidelines. Cells were subjected to flow cytometry using a BD LSRFortessa^TM^ cell analyzer system (BD Biosciences, Franklin Lakes, NJ, USA) and analyzed using FlowJo software (version 10; De Novo Software, Ashland, OR, USA).

### 2.4. siRNA Knockdown

RD cells were seeded at 0.25 × 10^6^ per well in 6-well plates, and after 24 h, growth media was replaced with 2 mL Accell delivery media supplemented with 2% FBS and 2 *μ*M siRNA against ezrin, radixin, moesin, or a combination of all three (Dharmacon, Lafayette, CO, USA). 2 *μ*M nontargeting scrambled siRNA was used as a negative control. After 48 h of incubation, 1 mL of fresh delivery media with 2 *μ*M siRNA was added to each well. At 96 h, viable cell counts were determined by trypan blue, and cells were lysed for Western blotting.

### 2.5. Western Blotting

Cells were lysed as previously described [27]. Protein concentration was estimated using the Pierce^TM^ BCA protein assay kit (ThermoFisher Scientific, Waltham, MA, USA). Protein lysates were separated by SDS-PAGE and subjected to Western blotting using the indicated primary antibodies (ezrin, radixin, moesin, phospho-ezrin (Thr567)/radixin (Thr564)/moesin (Thr558), cleaved caspase-3, and *β*-actin, 1 : 1000, Cell Signaling, Danvers, MA). HRP-linked goat *α*-rabbit IgG secondary antibody (1 : 10 000; Jackson ImmunoResearch, West Grove, PA, USA) and Amersham^TM^ ECL^TM^ Western blot detection reagents (GE Healthcare, Madison, WI, USA) were used to visualize protein bands. Relative band intensity was quantified using ImageJ software (NIH; https://imagej.nih.gov/ij/).

### 2.6. Xenograft Models

NOD-SCID-*γ*^−/−^ (NSG) mice were used as an immunocompromised murine model to enable successful propagation of RMS tumors in vivo. Subcutaneous (*n* = 12) and orthotopic (*n* = 20) tumors were developed in 6–8 weeks old female NSG mice by injecting 5 × 10^6^ RD cells suspended in Matrigel into bilateral flanks or bilateral biceps femoris muscles, respectively. Once tumors became palpable, mice were randomly divided into two treatment cohorts and received daily intraperitoneal administration of either vehicle control (DMSO) or 20 mg/kg body weight NSC668394 for subcutaneous and 40 mg/kg body weight NSC668394 for orthotopic models. Tumor dimensions were measured three times per week, and body weight was recorded once weekly. Treatment regimens were followed until the tumor burden in control mice was too large, and euthanasia was necessary. Tumors were measured by a digital caliper, and volumes were calculated as *V*=*W*^2^ × *L*/2 [28]. Animal studies were conducted in the Animal Tumor Core Facility of the Lerner Research Institute in accordance with guidelines approved by the Cleveland Clinic Institutional Animal Care and Use Committee.

### 2.7. Immunohistochemistry

Subcutaneous tumor xenografts were harvested postmortem, fixed in formalin, embedded in paraffin blocks, and sectioned at 5 *μ*m thickness. Degree of proliferation and apoptosis were measured using antibodies to Ki67 (Novus Biologicals; Centennial, CO) and cleaved caspase-3 (Cell Signaling, Danvers, MA). Immunohistochemistry staining was performed using the Discovery ULTRA automated stainer (Roche Diagnostics, Indianapolis, IN). In brief, antigen retrieval was performed using Discovery CC1 tris/borate/EDTA buffer (Roche Diagnostics, Indianapolis, IN), pH 8.0–8.5, for 56 minutes and 40 minutes, respectively, at 95°C. Slides were incubated with Ki67 antibody (1 : 1000) for 1 hour at room temperature and with cleaved caspase-3 antibody (1 : 200) for 40 min at room temperature. The antibodies were visualized using OmniMap anti-Rabbit HRP secondary (Roche Diagnostics, Indianapolis, IN) and the ChromoMap DAB detection kit (Roche Diagnostics, Indianapolis, IN). Last, the slides were counterstained with hematoxylin and bluing solution. Slides were imaged on a Leica SCN400 slide scanner (Leica Microsystems, Deerfield, Illinois, USA) and analyzed using Fiji image processing package of ImageJ [29]. To quantify the intensity of Ki67 and cleaved caspase-3 staining, images of DMSO or NSC668394-treated tumor sections were subjected to color deconvolution, which isolated the DAB staining mask from the hematoxylin counterstain. DAB masks were then converted to 8 bit, and five equally sized regions of interest (ROI) were selected. Mean grey values were measured in each ROI [30] and then converted to optical density (OD) values using the formula log_10_(255/mean grey value) as described in the ImageJ User Guide (https://imagej.nih.gov/ij/docs/user-guide-USbooklet.pdf) 

### 2.8. Statistical Analysis

All data were expressed as mean ± standard error of the mean (SEM). Statistical analyses were performed in GraphPad Prism 7 software (GraphPad Software Inc., La Jolla, CA, USA). Two-way analysis of variance (ANOVA) with Dunnett's multiple comparisons test was used to analyze cell viability, MTT, and annexin V/7-AAD data. One-way ANOVAs with Dunnett's or Holm-Šídák multiple comparisons were used to analyze cleaved caspase-3 Western blots and target expression in siRNA KD experiments, respectively. Tumor growth data were subject to repeated measures two-way ANOVA with Holm-Šídák multiple comparisons. The IC_50_ value for inhibition of cell metabolism was derived from a nonlinear regression on 96 h MTT data.

## 3. Results

### 3.1. NSC668394 Inhibits ERM Phosphorylation and Cell Viability in RMS Cell Lines

**Figure 1:**
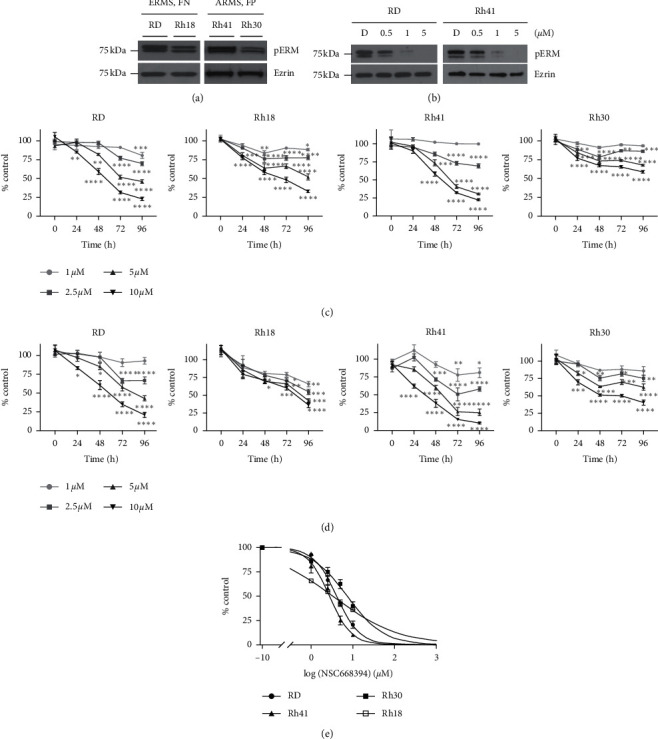
NSC668394 dephosphorylates ezrin and reduces cell viability and metabolism in RMS cells. (a) Western blot showing levels of phosphorylated ezrin-radixin-moesin (pERM) and total ezrin in untreated RMS cell lines. (b) Levels of pERM and total ezrin in RD and Rh41 whole cell lysates following treatment with indicated concentrations of NSC668394 or DMSO (D) for 1 h. (c) RMS cell viability following treatment with increasing concentrations of NSC668394 for 0–96 h as determined by trypan blue (d) RMS cell metabolism following treatment with increasing concentrations of NSC668394 as determined by MTT assay. (e) Variable slope nonlinear regression of 96 h MTT data was used to extrapolate IC50 values for inhibition of cell metabolism. All individual data points are represented as mean ± SEM and are representative of at least three independent experiments. Asterisks represent a significant difference between vehicle control DMSO and NSC668394 treated groups (^*∗*^*p* < 0.05, ^*∗∗*^*p* < 0.005, ^*∗∗∗*^*p* < 0.0005, and *p* < 0.0001=^*∗∗∗∗*^) for the given time point as determined by two-way ANOVA with Dunnett's multiple comparisons test.

Given that phosphorylated-ezrin (Thr567)-radixin (Thr564)-moesin (Thr558) levels determine the degree of activity in cells, the phosphorylation status of these proteins was examined in two ERMS subtype (RD and Rh18) and two ARMS subtype (Rh30 and Rh41) cell lines derived from different subtypes. RD and Rh18 are representative fusion-negative RMS tumors, whereas Rh30 and Rh41 are fusion-positive. Western blotting revealed that ERM proteins were constitutively phosphorylated at regulatory threonine residue in all RMS cell lines, regardless of subtype or fusion status (Figure 1(a)). However, RD and Rh41 showed the highest ERM phosphorylation and Rh30 the least (Figure 1(a)).

To examine whether exposure to NSC668394 would affect pERM levels, ERMS cell line RD and ARMS cell line Rh41 were treated with increasing concentrations of NSC668394 (0.5, 1, and 5 *μ*M) or vehicle control for 1 h. NSC668294 led to a dose-dependent reduction in pERM with marked suppression at concentrations greater than 0.5 *μ*M (Figure 1(b)). To investigate the impact of NSC668394 on cell viability, trypan blue assays were utilized. NSC668394 treatment effectively decreased RD, Rh18, Rh41, and Rh30 in a dose- and time-dependent manner, although Rh30 were affected the least (Figure 1(c)). To further assess effects on cellular metabolic activity as a measure of cell viability, MTT assays were performed. Following NSC668394 treatment, all four RMS cell lines showed a dose-dependent decrease in metabolic activity with trends similar to trypan blue-based viability measurement (Figure 1(d)). The IC_50_ for inhibition of cellular metabolism at 96 h was lowest for Rh41 (2.766 *μ*M), followed by that for Rh18 (3.291 *μ*M), RD (4.115 *μ*M), and Rh30 (7.338 *μ*M) (Figure 1(e)).

### 3.2. NSC668394 Induces Apoptosis in RMS Cells

**Figure 2:**
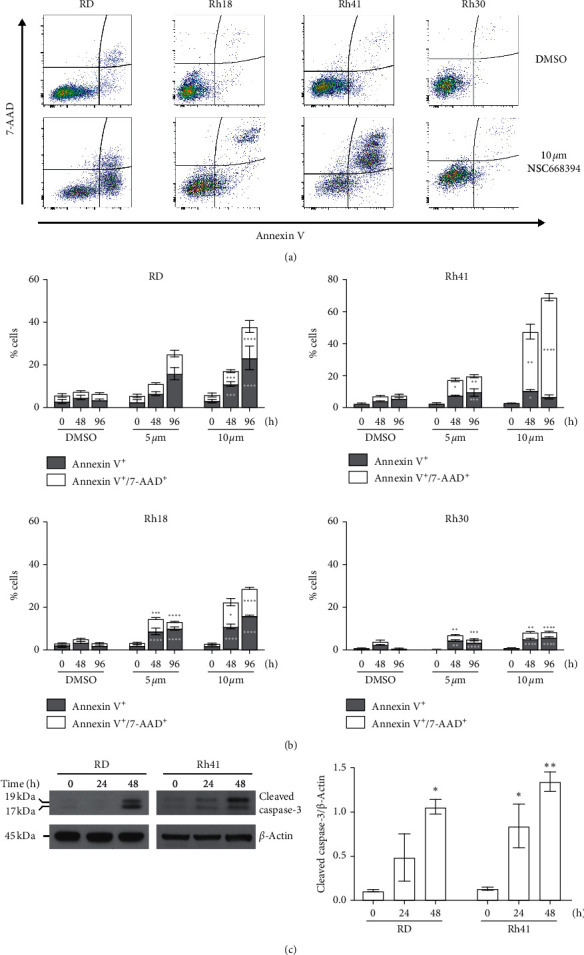
NSC668394 treatment triggers caspase-induced apoptosis in RMS cells. (a) Representative flow cytometry dot plots of RMS cell lines stained with annexin V and 7-AAD following treatment with 10 *μ*M NSC668394 or DMSO for 96 h. (b) Percentage of cells determined to be annexin V+ or annexin V+/7AAD+ were quantified after 0, 48, and 96 h after treatment with DMSO or NSC668394. Individual bars are represented as mean ± SEM and representative of three independent experiments. Asterisks represent a significant difference between vehicle and treated groups (^*∗*^*p* < 0.05, ^*∗∗*^*p* < 0.005, ^*∗∗∗*^*p* < 0.0005, and *p* < 0.0001=^*∗∗∗∗*^) for the given time point, as determined by two-way ANOVA with Dunnett's multiple comparisons test. (c) Representative Western blot of cleaved caspase-3 levels in RD or Rh41 cells treated with 10 *μ*M NSC668394 for indicated times. Relative band intensity was determined using ImageJ software and depicted in the bar graph. Blots are representative of three independent experiments.

To explore whether loss of cell viability was associated with induction of apoptosis, RMS cells were treated with increasing concentrations of NSC668394 for up to 96 h, stained with annexin V/7-AAD and analyzed by flow cytometry. Cells staining positive for annexin V but negative for 7-AAD were considered as early apoptotic, while those with positive staining for both annexin V and 7-AAD were qualified as late apoptotic or dead. Although all RMS cell lines had increased levels of early and late apoptotic cells with NSC668394 treatment, there was appreciable variability between cell lines similar to the variability observed for decrease in viability and metabolism (Figure 2(a)). ERMS cell line RD showed significant increases in the percentage of early and late apoptotic cells after 48 and 96 h with 10 *μ*M NSC668394 treatment with levels trending towards higher at 96 h at 5 *μ*M (Figure 2(b)). For ERMS line Rh18, levels of both early and late apoptosis were increased for both 5 and 10 *μ*M NSC68394 at 48 and 96 h. Despite this, the induction of apoptosis at 10 *μ*M treatment appeared to be lower in Rh18 compared to RD at 96 h. For ARMS cell line Rh41, 10 *μ*M NSC68394 treatment led to increased early and late apoptotic cells after 48 h and maintained elevated levels at 96 h, while 5 *μ*M treatment increased early and late apoptotic cells after 96 h. ARMS line Rh30 showed increases in early and late apoptosis at 48 and 96 h in 5 *μ*M and 10 *μ*M treated samples; however, the levels of apoptosis were drastically lower compared to that in the other three cell lines. To further confirm induction of apoptosis, RD and Rh41 cells were treated with 10 *μ*M NSC668394 and levels of caspase-3 cleavage detected by Western blot. Both RMS cell lines showed a significant induction of cleaved caspase-3 following NSC668394 treatment, with maximal increases observed at 48 h (Figure 2(c)).

### 3.3. siRNA Knockdown of ERM Proteins Reduces RMS Cell Viability

**Figure 3:**
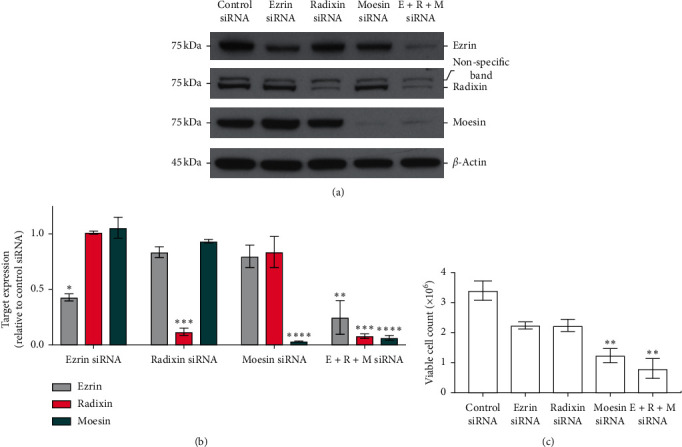
siRNA-mediated interference of ERM proteins reduces RMS cell viability. (a) Representative Western blots showing relative expression of ezrin, radixin, or moesin in RD cells treated with pooled siRNAs against ezrin, radixin, moesin, or all three together. Nontargeting scrambled (control) siRNA was used as a negative control for target protein expression, and *β*-actin was used as a loading control. (b) Quantification of ezrin, radixin, and moesin expression in cells treated with siRNA against ERM proteins relative to the corresponding target expression in control siRNA-treated cells. Expression is plotted as a function of relative band intensities determined using ImageJ software. (c) Viable cell counts as determined by trypan blue staining 96 h after treatment of RD cells with siRNA against ERM proteins or nontargeting control siRNA. Bars represent mean ± SEM, and asterisks represent a significant difference between control siRNA treatment group and ERM siRNA treatment groups as determined by one-way ANOVA with Dunnett's multiple comparisons tests (^*∗*^*p* < 0.05, ^*∗∗*^*p* < 0.005, ^*∗∗∗*^*p* < 0.0005, and *p* < 0.0001=^*∗∗∗∗*^). All data are representative of two independent experiments.

As NSC668394 treatment reduced the phosphorylation of all three ERM family proteins, we aimed to elucidate the individual contribution of ezrin, radixin, and moesin towards RMS cell viability. Accordingly, siRNA knockdown experiments were conducted in the RD cell line to examine the impact of suppressing ezrin, radixin, or moesin individually and all three ERM proteins together. RD cells were treated with each siRNA as described in methods. RD cells treated with siRNA against radixin and moesin achieved ∼90 and 95% decrease in expression, respectively (Figures 3(a) and 3(b)), whereas ezrin achieved only ∼50% decrease relative to the corresponding target expression in control siRNA-treated cells (Figures 3(a) and 3(b)). Simultaneous treatment of RD cells with siRNAs against all three ERM proteins resulted in ∼75% decrease in expression of ezrin, ∼90% for radixin, and ∼95 % for moesin (Figures 3(a) and 3(b)). At 96 h, viable cell counts were determined by trypan blue. When compared to control siRNA-treated (∼3.4 × 10^6^ cells) RD cells, ezrin and radixin knockdown both showed reduced cell viability (∼2.24 × 10^6^ cells) (Figure 3(c)). Moesin KD had the greatest impact on cell viability (∼1.24 × 10^6^ cells) amongst individually knocked down ERM proteins (Figure 3(c)). Unsurprisingly, combined suppression of all three ERM proteins had the greatest impact on RMS cell viability (∼0.8 × 10^6^ cells) at 96 h (Figure 3(c)).

### 3.4. NSC668394 Decreases RD Tumor Growth In Vivo

**Figure 4:**
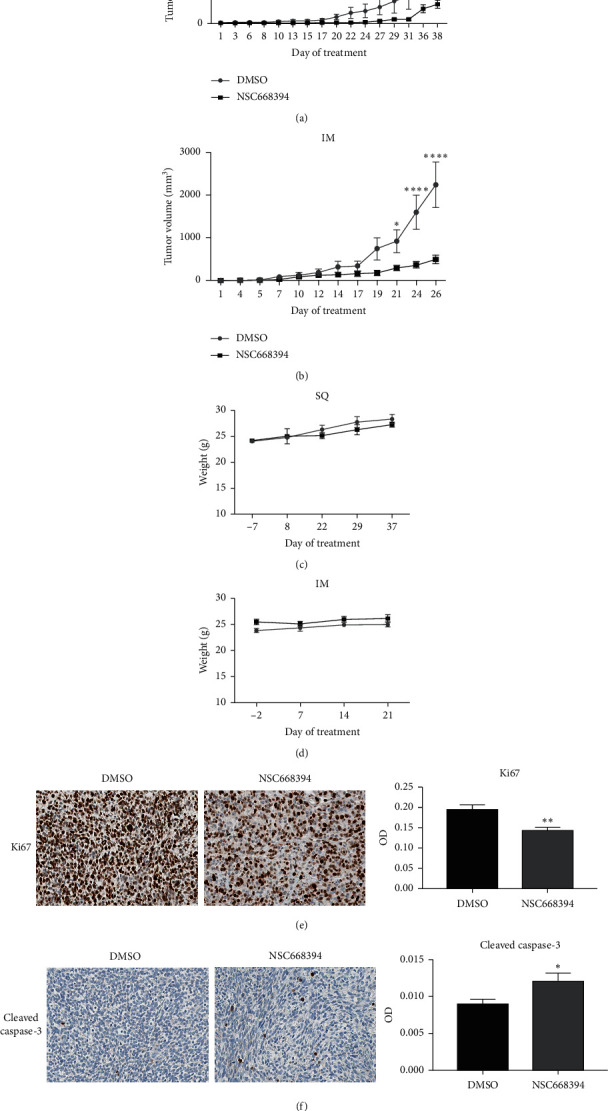
NSC668394 inhibits the growth of RD xenograft in vivo. (a) Subcutaneous (SQ) RD tumor volumes in NSG mice subjected to daily intraperitoneal administration of either 20 mg/kg NSC668394 (*n* = 6) or DMSO (*n* = 6). (b) Orthotopic intramuscular (IM) RD tumor volumes in NSG mice subjected to daily intraperitoneal administration of 40 mg/kg NSC668394 (*n* = 10) or DMSO (*n* = 10). Total body weight of NSG mice bearing subcutaneous (c) and orthotopic (d) RD tumors on the indicated days. Negative values shown on the *x*-axis of total body weight plots represent days prior to starting NSC668384 treatment. Individual data points are represented as mean ± SEM. Asterisks represent a significant difference between treatment groups (^*∗*^*p* < 0.05, ^*∗∗*^*p* < 0.005, ^*∗∗∗*^*p* < 0.0005, and *p* < 0.0001=^*∗∗∗∗*^) for the given time point as determined by RM two-way ANOVA with Holm-Šídák multiple comparisons tests. Representative images of subcutaneous xenograft sections stained for Ki67 (e) or cleaved caspase-3 (f). Each stain was quantified as optical density using Fiji extension of ImageJ and plotted as bar graphs. Bars represent mean ± SEM, and asterisks represent a significant difference between treatment groups as determined by the unpaired *t*-test (^*∗*^*p* < 0.05 and ^*∗∗*^*p* < 0.005).

We next examined if NSC668394 would alter the growth of human RMS cell line-derived tumors in vivo. Initial effects of NSC663894 on tumor growth were modeled using subcutaneous (SQ) xenografts (*n* = 12) of the RD cell line in NSG mice. Once tumors were palpable, mice were subjected to daily intraperitoneal (IP) administration of either vehicle control, DMSO (*n* = 6 tumors), or 20 mg/kg body weight NSC668394 (*n* = 6 tumors). NSC668394 administration significantly decreased the growth of RD tumors (Figure 4(a)). The mean SQ tumor volume in the NSC68394-treated cohort (*x̅* = 216.8 mm^3^) was significantly lower than the vehicle control (*x̅* = 747.7 mm^3^) cohort at day 36 (*p*=0.0033), and this trend of lower relative volume was maintained through subsequent measured time points (Figure 4(a)). To examine the effects of NSC668394 in an orthotopic model, intramuscular (IM) RD xenografts (*n* = 20) were also established in NSG mice. To account for the IM tumors being sequestered within the muscle, NSC668394 dosage was escalated to 40 mg/kg body weight to ensure that adequate levels of NSC668394 were reaching the tumors. Following treatment, IM tumor volume in the NSC68394-treated cohort (*n* = 10 tumors, *x̅* = 291.3 mm^3^) was significantly lower than vehicle control-treated cohort (*n* = 10 tumors, *x̅* = 914.2 mm^3^) beginning at day 21 of treatment (*p*=0.0404), and this relative decrease in volume persisted through remaining measured time points (Figure 4(b)). Treatment regimens for SQ and IM xenografts were followed for 38 days and 26 days, respectively, at which point, the tumor burden in vehicle control-treated mice was too large, and euthanasia was necessary. Mean body weight was compared between treatment cohorts as an indicator of systemic toxicity. In both SQ (Figure 4(c)) and IM (Figure 4(d)) xenograft models, NSC668394-treated mice did not exhibit any significant alterations in body weight as compared to the vehicle control cohort at any measured time point. No other signs of morbidity or systemic toxicity, such as changes in grooming behavior, were recognized in either cohort. To investigate underlying causes of decreased tumor growth, subcutaneous xenografts were harvested at the day of euthanasia, fixed in formalin, and embedded in paraffin blocks for sectioning. Sections from each cohort were stained for Ki67 or cleaved caspase-3 as markers of proliferation and apoptosis, respectively. Quantitative image analysis of NSC668394-treated tumors sections (*n* = 5) showed decreased Ki67 (*p* < 0.01) (Figure 4(e)) and increased cleaved caspase-3 (*p* < 0.05) (Figure 4(f)) staining when compared to DMSO-treated tumor sections (*n* = 5).

## 4. Discussion

Despite ezrin being cited as an important driver of progression and metastasis in RMS, neither the effects of its pharmacological inhibition nor the contribution of other ERM family members radixin and moesin has previously been considered [18]. We aimed to uncover the potential therapeutic properties of inhibiting ERM proteins in RMS. Here, we demonstrate the importance of ERM proteins in RMS growth using cell culture and xenograft models and build support for ERM proteins as therapeutically actionable targets in RMS.

Pioneering research on the therapeutic benefits of ezrin inhibition in osteosarcoma (OS) initially identified two small molecules, NSC50787 and NSC668394, which both dephosphorylate ezrin at its Thr567 residue, leading to inactivation [23, 31]. To date, NSC668394 has been reported to display anticancer activity in a variety of tumors including, OS, diffuse large B-cell lymphoma (DLBCL), glioblastoma, and breast cancer [21, 23, 32, 33]. However, no prior studies had investigated the effects of small molecule inhibition of ERM proteins on RMS cell viability, metabolism, or apoptosis. Our in vitro assays revealed that NSC668394 effectively reduced RMS cell viability and metabolism in a dose-dependent manner with an IC_50_ ranging from 2.766–7.338 *μ*M. Cell lines representing both ERMS and ARMS responded to NSC668394 treatment, highlighting the importance of ERM proteins to the growth and survival of RMS regardless of histological subtype or PAX3/7-fusion status. It is also worth noting that these findings are consistent with those documented in DLBCL and OS, which reported similar IC_50_ values of 5.8 *μ*M and 5.9 *μ*M, respectively [32, 34]. Furthermore, we showed that inhibition of ERM proteins effectively reduced RMS viability through induction of apoptosis. As with the observed decreases in cell viability and metabolism, the degree to which ERM inhibition triggered apoptosis was variable between cell lines and did not seem to depend on subtype or PAX3/7-fusion status. Overall, our results demonstrate the ability of a small molecule inhibitor of ERM phosphorylation to display tumor suppressive activity in RMS. In vivo ERM inhibition in two xenograft models confirmed its tumor suppressive characteristics in a physiologically relevant setting. Moreover, IHC revealed decreased Ki67 and increased cleaved caspase-3 staining of NSC668394-treated tumor sections, which supports the antiproliferative and apoptosis-inducing characteristics of ERM inhibition in vivo. Furthermore, the lack of weight loss in mice treated with NSC668394 indicates a favorable safety profile, suggesting that it can affect RMS tumor growth without presenting debilitating systemic side effects.

The interpretation of our findings relies on the specificity of NSC668394. Phosphorylation of ERM proteins can be regulated by multiple kinases, including PKC [20, 35–37]. Therefore, previous studies considered two mechanism of NSC668394 activity—direct binding of ezrin and prevention of phosphorylation or inhibition of kinases, which typically phosphorylates ezrin. Bulut et al. showed that NSC668394 did not have a significant inhibitory effect on PKC*α*, PKC*γ*, or PKC*ι* at concentrations up to 100 *μ*M and reported a much weaker binding affinity to PKC*ι* (KD = 58.1 *μ*M) as compared to ezrin (*K*_D_ = 12.59) [23]. Moreover, our previous study in DLBCL showed that overexpression of ezrin in cells treated with NSC668394 greatly diminishes its ability to decrease cell viability, supporting the specificity of NSC668394 to ezrin and its highly homologous family members [32]. Similarly, another study that reported antimetastatic properties of NSC668394 in breast cancer showed that ezrin-deficient cells treated with NSC668394 no longer showed reduced migration [38]. Several of our findings also support the specificity of NSC668394. The efficacy of NSC668394 in reducing RMS cell viability and inducing apoptosis seemed to correlate with the level of basal pERM in resting RMS cell lines. RD and Rh41 had relatively higher levels of pERM in resting cells and showed greater decrease in cell viability with larger increase in apoptosis, while Rh18 and Rh30 had relatively lower levels of pERM and experienced relatively less impact of NSC668394 on viability and apoptosis. As previous studies were mainly focused on ezrin being a driver in RMS, we were interested in whether the observed impacts to cell survival were solely due to interrupted ezrin function or if radixin and moesin also contributed to RMS survival. Interestingly, siRNA knockdown of ERM proteins revealed that all three regulate RMS survival to some extent. While radixin and moesin siRNAs achieved a nearly complete knockdown, ezrin was only partially knocked down, which suggests that it may contribute less to cell growth than to metastasis. Moesin knockdown resulted in the largest individual impact on survival, with ezrin and radixin reducing cell viability to a lesser extent. However, the largest impact on cell viability was observed when all three ERM proteins were simultaneously knocked down. Together, these findings suggest that the effects of NSC6683894 are likely a consequence of suppressed ezrin, radixin, and moesin function as opposed to off-target effects of the small molecule.

Although the current study focuses largely on ERM protein's role in RMS survival and apoptosis, it is important to consider the well-established role of ERM proteins as prometastatic molecules. Interrupting the function of ERM-family proteins has been shown to affect migration in various other tumor types. For instance, ezrin inhibition reduced metastatic spread of OS and breast cancer, while silencing moesin and radixin were shown to reduce migration and invasion of melanoma and colon cancer, respectively. In RMS, metastasis is most commonly associated with the ARMS subtype due to its notably higher aggressiveness and migratory potential. To this end, future studies will investigate the impact of ERM inhibition on RMS metastasis.

Standard chemotherapy regimens for RMS commonly include vincristine, actinomycin-D, and cyclophosphamide (VAC) combinational therapy, providing a 5-year survival of ∼70–90% for low-risk patients [15, 39]. However, the survival rate sharply decreases in intermediate and high-risk patients as the chemotherapy regimen becomes more intensive, and additional adjuvants are often included, thereby highlighting the necessity for innovations to current treatment regimens [15, 39, 40]. Accordingly, the use of pharmacological ERM inhibitors may deserve realistic consideration in the future as an adjuvant to standard chemotherapies for RMS.

As conclusions of this study, we provide evidence that pharmacologic inhibition of ERM proteins by the small molecule NSC668394 induces loss of RMS growth in preclinical cell culture and xenograft models. Collectively, our study suggests that ERM proteins may be clinically actionable targets in RMS.

## Data Availability

All the data used to support the findings of this study are available from the corresponding author upon request.

## Abbreviations

ERMS: Embryonal rhabdomyosarcoma
ARMS: Alveolar rhabdomyosarcoma
ERM: Ezrin-Radixin-Moesin.
